# A New Data-Driven Control System for MEMSs Gyroscopes: Dynamics Estimation by Type-3 Fuzzy Systems

**DOI:** 10.3390/mi12111390

**Published:** 2021-11-12

**Authors:** Khalid A. Alattas, Ardashir Mohammadzadeh, Saleh Mobayen, Ayman A. Aly, Bassem F. Felemban, Mai The Vu

**Affiliations:** 1 Department of Computer Science and Artificial Intelligence, College of Computer Science and Engineering, University of Jeddah, Jeddah 23890, Saudi Arabia; kaalattas@uj.edu.sa; 2 Independent Researcher, Baku 1148, Azerbaijan; 3 Future Technology Research Center, National Yunlin University of Science and Technology, Douliu 64002, Taiwan; 4 Department of Mechanical Engineering, College of Engineering, Taif University, P.O. Box 11099, Taif 21944, Saudi Arabia; aymanaly@tu.edu.sa (A.A.A.); b.felemban@tu.edu.sa (B.F.F.); 5 School of Intelligent Mechatronics Engineering, Sejong University, Seoul 05006, Korea

**Keywords:** fuzzy system, learning algorithm, MEMS gyroscopes, machine learning, LMI set, data-driven control

## Abstract

In this study, a novel data-driven control scheme is presented for MEMS gyroscopes (MEMS-Gs). The uncertainties are tackled by suggested type-3 fuzzy system with non-singleton fuzzification (NT3FS). Besides the dynamics uncertainties, the suggested NT3FS can also handle the input measurement errors. The rules of NT3FS are online tuned to better compensate the disturbances. By the input-output data set a data-driven scheme is designed, and a new LMI set is presented to ensure the stability. By several simulations and comparisons the superiority of the introduced control scheme is demonstrated.

## 1. Introduction

Micro-electro-mechanical-system gyroscopes (MEMS-Gs) have specific properties and vast applications in navigation systems, industrial plants, automobile systems, and so on. MEMS-Gs mainly measure the rate of the rotation around the axis. The control problem of MEMS-Gs can be challengeable, because the MEMS-Gs performance can be significantly influenced by fundamental perturbations such as time-varying dynamics, cross stiffness, external noises, and damping [1,2,3,4].

Interestingly, design of MEMS-Gs can be impressively boosted through powerful control schemes which can eliminate or at least reduce the error signals [5,6]. In turn, some algorithms have been proposed to control MEMS-Gs. For instance, the chattering phenomenon in control signal applied to the MEMS-Gs has been removed by employing an integral sliding-mode control (SMC) [7]. Moreover, a robust SMC has been developed to improve trajectory tracking and remove the chattering for the similar problem [8]. An adaptive dynamic SMC with the sliding surface comprised of fractional-order terms has been utilized to control the *Z*-axis vibrating MEMS-Gs in [9], and an adaptive dynamic SMC via a backstepping technique to analyze the same problem has been used in [10]. Based on an LMI approach and the concept of a nonsingular terminal SMC strategy, the stabilization conditions of the MEMS-Gs have been addressed in [11]. The tracking problem of uncertain MEMS-Gs with disturbances has been analyzed by using an adaptive prescribed performance SMC in [12]. Moreover, the nonsingular terminal SMC which involves an online identifier to approximate the angular velocity has been suggested by using the backstepping scheme in [13].

On the other hand, the stability analysis and control problem become more complex when unknown nonlinear dynamics and uncertainties exist in the model of MEMS-Gs. These types of uncertainties may devastate the performance of MEMS-Gs [14].

In the literature, FLS-based control techniques are widely used for uncertain systems [15,16]. For instance, in [17], a type-2 (T2) FLS with a tuned scheme for secondary memberships has been implemented for the frequency regulation problem subject to unknown dynamics and multiple disturbances. In [18], based on T2-FLSs, the unknown nonlinearities have been approximated to analyze the synchronization of chaotic systems under unknown slave systems. The problem of synchronization of chaotic systems in the presence of unknown dynamics has been solved by using a dynamic programming technique and type-2 wavelet-based FLSs [19]. To upgrade the estimation precision in the system identification problem, a dynamic deep learned T2-FLS has been introduced in [20]. The predictive control of glucose level with unknown metabolism has been studied by using T2-FLSs in [21]. Based on an LMI scheme and a deep tuned T2-FLS, the leader following problem of multi-agent plants under perturbed dynamics has been studied in [22]. Moreover, to upgrade approximation performance, an interval type-3 (T3) FLS with an online learning scheme has been developed in [23]. Recently, a self-organizing interval T3-FLS has been suggested in [24] to promote the precisions of a learning algorithms in versus of non-Gaussian noises. In [25] a new FLS based predictive control system is introduced for networked systems. In [26], a new system based on FLSs is designed for transporting of hazardous materials.

These techniques have provided the opportunities to address the uncertainties and unknown dynamics of MEMS-Gs. A fractional-order SMC of a micro gyroscope with unknown model has been studied via a double-loop FLS [27]. The bat algorithm has been used for parameter tuning of PID SMC of MEMS-Gs in [28]. Furthermore, both the SMC and the NTSMC have been designed by considering composite neural learning method to achieve finite-time stability and enhance the tracking accuracy of MEMS-Gs [29]. Authors in [30] have utilized a neural adaptive control with hysteresis logarithmic quantizer for the MEMS-GS in the presence of constraints on the states. In [31], using a simple neural network (NN) to approximate nonlinearity of the MEMS-Gs, the fast terminal SMC has been developed to analyze short-time convergence. Based on the error compensation scheme, the TSMC problem has been investigated for the subject of uncertainties in [32]. An output feedback controller has been designed using NNs and SMC for the same problem in [33]. Moreover, a NN constrained output feedback controller which incorporates a hybrid quantizer has been suggested to enhance tracking error of the MEMS-Gs [34]. Recently, the predictive control (MPC) approach with T3FLSs has been designed to control MEMS-Gs which suffers from actuator faults [35]. Considering non-singleton FLSs and Boltzmann machine, a new fuzzy controller has been suggested for MEMS-Gs in the presence of nonlinearities and unknown dynamics [36].

Data-based control strategies are at the center of attention due to their applications in engineering. Along with model-based and system identification approaches, direct data-driven control methods can be implemented to investigate the stability analysis of systems with unknown dynamics. In this regard, direct data-driven control method is able to tackle the complexities of learning a precise model of a system. Recently, some valuable direct data-driven control techniques have been proposed. For instance, based on persistently exciting data, data-dependent LMIs have been provided in [37,38] to obtain the optimal control and robustness in the presence of noise corrupted measurements via applying a data-based state feedback controller. Authors in [39] propose a method to improve the conditions of designing data-driven control. Based on past measured trajectories, an implicit model has been considered to design a robust data-driven MPC [40]. Recently, these ideas have also been utilized to control the unknown nonlinear polynomial systems [41]. Utilizing the framework of matrix S-Lemma and LMIs, $H_{2}$ and $H_{\infty }$ data-based controllers are designed through noisy data in [42]. Based on data-driven control and reinforcement learning, the Linear Quadratic Regulator (LQR) has been developed for the stabilization of linear systems with unknown dynamics in [43]. Data-driven control and fault estimation of unknown systems has been studied in [44]. Moreover, a data-based feedback controller with a guaranteed attraction has been considered to analyze the stabilization of bilinear systems [45].

Motivated by above research studies, in this paper, a non-singleton T3-FLS is established to tackle the nonlinearities of the system model and unknown dynamics of MEMS-Gs, which in turn enables us to develop the accuracy of the suggested controller. The contributions of this paper are consist of developing the direct data-driven control strategy and merging with a non-singleton type-3 FLS to design a novel hybrid controller. Based on the finite response samples and considering the basic sufficient conditions, a hybrid controller is utilized for the stability of the tracking error. In contrast to the proposed methods of [35,36], where the MPC strategy and a deep learned RBM have been utilized, in current study, a data-driven scheme is implemented to upgrade the accuracy of the applied controller, characterize, and achieve the stability of the error system without explicitly identifying a model or using a deep learned RBM. More specifically, to tackle the conditions on characterization of the error dynamic system proposed in [35,36] which lead to the conservatism, modeling error, and high computational complexity, direct data-driven control mechanism is formulated to enhance the system performance. To this goal, based on the optimization problem, sufficient data-based conditions in the framework of the LMI are acquired and the gain of the data-based controller is computed. Employing the suggested direct data-driven control method, the stability of the error dynamics is ensured via a Lyapunov function, which in turn ensures that our data-driven algorithm steers the error signal to the zero.

## 2. Problem Formulation

Consider the following dynamics of MEMS-G [35]:

(1) $$\begin{array}{cc} \; & M{\ddot{\tilde{r}}}_{1}+a_{11}{\dot{\tilde{r}}}_{1}+\left(a_{12}-2M{\tilde{\theta }}_{\rho }\right){\dot{\tilde{r}}}_{2}+\left(\Lambda_{11}-M{{\tilde{\theta }}_{\rho }}^{2}\right){\dot{\tilde{r}}}_{1}+\Lambda_{11}{\tilde{r}}_{2}+\Lambda_{13}{\tilde{r}}_{1}^{3}={\tilde{\eta }}_{1} \end{array}$$

(2) $$\begin{array}{cc} \; & M{\ddot{\tilde{r}}}_{2}+a_{22}{\dot{\tilde{r}}}_{1}+\left(a_{21}+2M{\tilde{\theta }}_{\rho }\right){\dot{\tilde{r}}}_{1}+\left(\Lambda_{22}-M{{\tilde{\theta }}_{\rho }}^{2}\right){\dot{\tilde{r}}}_{2}+\Lambda_{21}{\tilde{r}}_{1}+\Lambda_{23}{\tilde{r}}_{2}^{3}={\tilde{\eta }}_{2} \end{array}$$

where $\Lambda_{13},\Lambda_{21},\Lambda_{23}$, and $\Lambda_{11},\Lambda_{22}$ are the stiffness coefficients and the coefficients of stiffness coupling. Moreover, ${\tilde{r}}_{1}$/${\tilde{r}}_{2}$ denotes the displacement of drive/sensitive axis and ${\tilde{\theta }}_{\rho }$ is the level sensitivity. The dimensionless positions are as follows:

(3) $$\begin{array}{cc} \; & M{\ddot{r}}_{1}+a_{11}{\dot{r}}_{1}+\left(a_{12}-2M\theta_{\rho }\right){\dot{r}}_{2}+\left(\Lambda_{11}-M\theta_{\rho }^{2}\right){\dot{r}}_{1}+\Lambda_{11}r_{2}+\Lambda_{13}r_{1}^{3}=\eta_{1} \end{array}$$

(4) $$\begin{array}{cc} \; & M{\ddot{r}}_{2}+a_{22}{\dot{r}}_{1}+\left(a_{21}+2M\theta_{\rho }\right){\dot{r}}_{1}+\left(\Lambda_{22}-M\theta_{\rho }^{2}\right){\dot{r}}_{2}+\Lambda_{21}r_{1}+\Lambda_{23}r_{2}^{3}=\eta_{2} \end{array}$$

One can estimate the dynamics of MEMS-G as follows:

(5) $$\begin{array}{cc} \; & {\dot{\hat{r}}}_{1}=G_{1}\left(x|\Xi_{r_{1}}\right)+\eta_{1} \end{array}$$

(6) $$\begin{array}{cc} \; & {\dot{\hat{r}}}_{2}=G_{2}\left(x|\Xi_{r_{2}}\right)+\eta_{2} \end{array}$$

where $G_{1}\left(x|\Xi_{r_{1}}\right)$ and $G_{1}\left(x|\Xi_{r_{1}}\right)$ are the designed NT3FSs, $\Xi_{r_{1}}$ and $\Xi_{r_{1}}$ are the tunable parameters and the vector *x* is:

(7) $$\begin{array}{c} x={\left[\begin{array}{cccc} r_{1} & r_{2} & {\dot{r}}_{1} & {\dot{r}}_{2} \end{array}\right]}^{T} \end{array}$$

**Figure 1:**
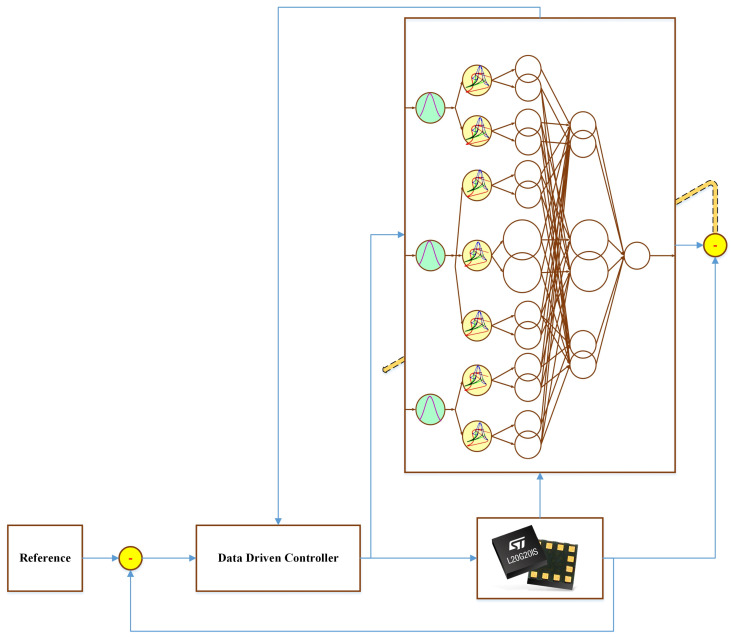
General control scheme.

**Figure 2:**
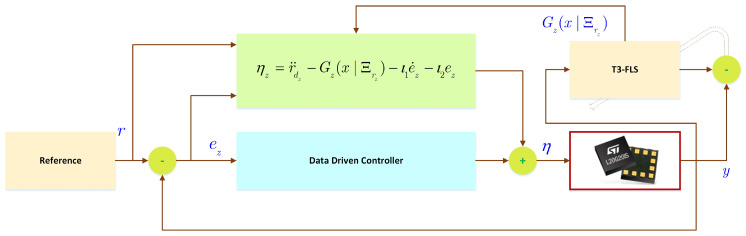
Control scheme.

By applying the online optimized FLS to tackle the uncertainties, a data-drive scheme is proposed stabilize the tracking error dynamics. See the general scheme in Figure 1 and Figure 2.

## 3. Type-3 FLS

**Figure 3:**
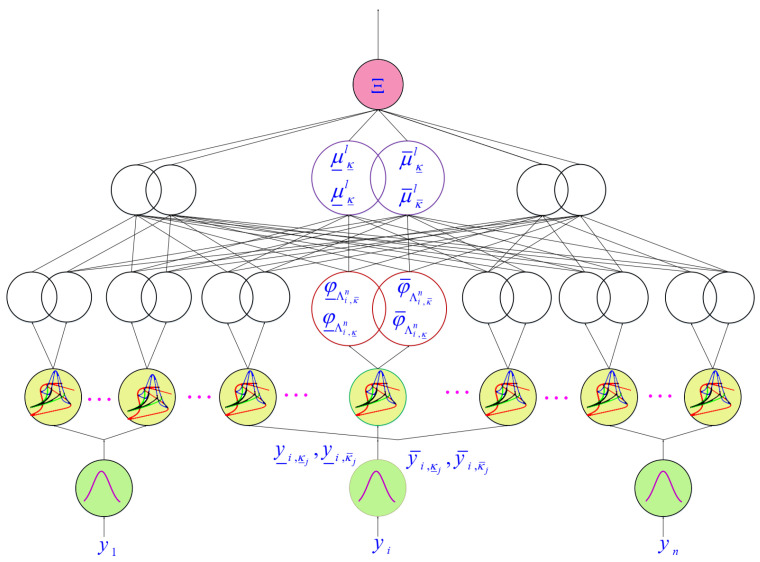
NT3FS structure.

The dynamics of the MEMS-G are perturbed by various disturbances and also the mathematical equations are considered to be fully uncertain. Then in control of MEMS-Gs we deal with a high uncertain problem. Type-3 FLSs can better model the high-level uncertainties, because of their higher degrees of freedom in contrast to type-1 and type-2 counter parts. The secondary memberships and upper bounds of uncertainties in type-3 fuzzy sets are not fixed values but they are also fuzzy sets. Regarding above reasons, in this paper T3-FLSs are employed to tackle the uncertainties in dynamics of MEMS-G as given in (5). The suggested scheme is given in Figure 3. The computation details of output signal is written as:

(1) The inputs of NT3FSs are $x=\left[y_{1},{\dot{y}}_{1},y_{2},{\dot{y}}_{2}\right]$.

(2) The operation of fuzzifications are applied as:

(8) $${\bar{x}}_{i,{\bar{\kappa }}_{j}}\left(t\right)=\frac{x_{i}\left(t\right){\bar{\sigma }}_{\Lambda_{i}^{n},{\bar{\kappa }}_{j}}^{2}+c_{\Lambda_{i}^{n},{\bar{\kappa }}_{j}}{\bar{\sigma }}_{x}^{2}}{{\bar{\sigma }}_{\Lambda_{i}^{n},{\bar{\kappa }}_{j}}^{2}+{\bar{\sigma }}_{x}^{2}},$$

(9) $${\bar{x}}_{i,{\underline{\kappa }}_{j}}\left(t\right)=\frac{x_{i}\left(t\right){\bar{\sigma }}_{\Lambda_{i}^{n},{\underline{\kappa }}_{j}}^{2}+c_{\Lambda_{i}^{n},{\underline{\kappa }}_{j}}{\bar{\sigma }}_{x}^{2}}{{\bar{\sigma }}_{\Lambda_{i}^{n},{\underline{\kappa }}_{j}}^{2}+{\bar{\sigma }}_{x}^{2}},$$

(10) $${\underline{x}}_{i,{\bar{\kappa }}_{j}}\left(t\right)=\frac{x_{i}\left(t\right){\underline{\sigma }}_{\Lambda_{i}^{n},{\bar{\kappa }}_{j}}^{2}+c_{\Lambda_{i}^{n},{\bar{\kappa }}_{j}}{\bar{\sigma }}_{x}^{2}}{{\underline{\sigma }}_{\Lambda_{i}^{n},{\bar{\kappa }}_{j}}^{2}+{\bar{\sigma }}_{x}^{2}},$$

(11) $${\underline{x}}_{i,{\underline{\kappa }}_{j}}\left(t\right)=\frac{x_{i}\left(t\right){\underline{\sigma }}_{\Lambda_{i}^{n},{\underline{\kappa }}_{j}}^{2}+c_{\Lambda_{i}^{n},{\underline{\kappa }}_{j}}{\bar{\sigma }}_{x}^{2}}{{\underline{\sigma }}_{\Lambda_{i}^{n},{\underline{\kappa }}_{j}}^{2}+{\bar{\sigma }}_{x}^{2}},$$

where, $x_{i}\left(t\right)$ is the *i*-th input, $c_{\Lambda_{i}^{n},{\bar{\kappa }}_{j}}$, ${\underline{\sigma }}_{\Lambda_{i}^{n},{\underline{\kappa }}_{j}}$, ${\underline{\sigma }}_{\Lambda_{i}^{n},{\bar{\kappa }}_{j}}$, ${\bar{\sigma }}_{\Lambda_{i}^{n},{\underline{\kappa }}_{j}}$, ${\bar{\sigma }}_{\Lambda_{i}^{n},{\bar{\kappa }}_{j}}$ denote the mean and standard-divisions for n-th fuzzy set (FS) for $y_{i}$ at κ slice level. ${\bar{\sigma }}_{x}$ is a constant value.

(3) The memberships are written as:

(12) $${\bar{\varphi }}_{\Lambda_{i}^{n},{\bar{\kappa }}_{j}}\left(x_{i}\left(t\right)\right)=\exp\left(-\frac{\left({\bar{x}}_{i,{\bar{\kappa }}_{j}}\left(t\right)-c_{\Lambda_{i}^{n},{\bar{\kappa }}_{j}}\right)}{{\bar{\sigma }}_{\Lambda_{i}^{n},{\bar{\kappa }}_{j}}^{2}}\right),$$

(13) $${\bar{\varphi }}_{\Lambda_{i}^{n},{\underline{\kappa }}_{j}}\left(x_{i}\left(t\right)\right)=\exp\left(-\frac{\left({\bar{x}}_{i,{\underline{\kappa }}_{j}}\left(t\right)-c_{\Lambda_{i}^{n},{\underline{\kappa }}_{j}}\right)}{{\bar{\sigma }}_{\Lambda_{i}^{n},{\underline{\kappa }}_{j}}^{2}}\right),$$

(14) $${\underline{\varphi }}_{\Lambda_{i}^{n},{\bar{\kappa }}_{j}}\left(x_{i}\left(t\right)\right)=\exp\left(-\frac{\left({\underline{x}}_{i,{\bar{\kappa }}_{j}}\left(t\right)-c_{\Lambda_{i}^{n},{\bar{\kappa }}_{j}}\right)}{{\underline{\sigma }}_{\Lambda_{i}^{n},{\bar{\kappa }}_{j}}^{2}}\right),$$

(15) $${\underline{\varphi }}_{\Lambda_{i}^{n},{\underline{\kappa }}_{j}}\left(x_{i}\left(t\right)\right)=\exp\left(-\frac{\left({\underline{x}}_{i,{\underline{\kappa }}_{j}}\left(t\right)-c_{\Lambda_{i}^{n},{\underline{\kappa }}_{j}}\right)}{{\underline{\sigma }}_{\Lambda_{i}^{n},{\underline{\kappa }}_{j}}^{2}}\right).$$

(4) The firing level of rules are [23]:

(16) $${\bar{\mu }}_{{\bar{\kappa }}_{j}}^{h}={\bar{\varphi }}_{\Lambda_{1}^{n_{1}},{\bar{\kappa }}_{j}}\;\cdot \;{\bar{\varphi }}_{\Lambda_{2}^{n_{2}},{\bar{\kappa }}_{j}}\ldots {\bar{\varphi }}_{\Lambda_{n}^{n_{N}},{\bar{\kappa }}_{j}},$$

(17) $${\bar{\mu }}_{{\underline{\kappa }}_{j}}^{h}={\bar{\varphi }}_{\Lambda_{1}^{n_{1}},{\underline{\kappa }}_{j}}\;\cdot \;{\bar{\varphi }}_{\Lambda_{2}^{n_{2}},{\underline{\kappa }}_{j}}\ldots {\bar{\varphi }}_{\Lambda_{n}^{n_{N}},{\underline{\kappa }}_{j}},$$

(18) $${\underline{\mu }}_{{\bar{\kappa }}_{j}}^{h}={\underline{\mu }}_{\Lambda_{1}^{n_{1}},{\bar{\kappa }}_{j}}\;\cdot \;{\underline{\mu }}_{\Lambda_{2}^{n_{2}},{\bar{\kappa }}_{j}}\ldots {\underline{\mu }}_{\Lambda_{n}^{n_{N}},{\bar{\kappa }}_{j}},$$

(19) $${\underline{\mu }}_{{\underline{\kappa }}_{j}}^{h}={\underline{\mu }}_{\Lambda_{1}^{n_{1}},{\underline{\kappa }}_{j}}\;\cdot \;{\underline{\mu }}_{\Lambda_{2}^{n_{2}},{\underline{\kappa }}_{j}}\ldots {\underline{\mu }}_{\Lambda_{n}^{n_{N}},{\underline{\kappa }}_{j}}.$$

where, *N* is input numbers. The *h*-th rule is given as:

(20) $$\begin{array}{c} y_{1}\;\mathrm{is}\;{\bar{\varphi }}_{\Lambda_{1}^{n_{1}},{\bar{\kappa }}_{j}}\;\;\mathrm{and}\;\;y_{2}\;\mathrm{is}\;\;\;{\bar{\varphi }}_{\Lambda_{2}^{n_{2}},{\bar{\kappa }}_{j}}\;\;\mathrm{and}\;\;\\ y_{n}\;\mathrm{is}\;{\bar{\varphi }}_{\Lambda_{n}^{n_{N}},{\bar{\kappa }}_{j}}\;\;\mathrm{Then}\;\;y_{h}\;\in \;\left[{\underline{\Xi }}_{h,j},{\bar{\Xi }}_{h,j}\right], \end{array}$$

where, ${\underline{\Xi }}_{h,j}$ and ${\bar{\Xi }}_{h,j}$ denote rule coefficients.

(5) The output signal is written as [23]:

(21) $$\begin{array}{c} y=\frac{\sum_{j=1}^{n_{\kappa }}}{\sum_{j=1}^{n_{\kappa }}{\bar{\kappa }}_{j}}\left(\frac{\sum_{h=1}^{Y}{\bar{\kappa }}_{j}\left({\bar{\mu }}_{{\bar{\kappa }}_{j}}^{h}+{\underline{\mu }}_{{\bar{\kappa }}_{j}}^{h}\right){\bar{\Xi }}_{h,j}/2}{\sum_{h=1}^{Y}\left({\bar{\mu }}_{{\bar{\kappa }}_{j}}^{h}+{\underline{\mu }}_{{\bar{\kappa }}_{j}}^{h}\right)}+\right.\left.\frac{{\underline{\kappa }}_{j}\sum_{h=1}^{Y}\left({\bar{\mu }}_{{\underline{\kappa }}_{j}}^{h}+{\underline{\mu }}_{{\underline{\kappa }}_{j}}^{h}\right){\underline{\Xi }}_{h,j}/2}{\sum_{h=1}^{Y}\left({\bar{\mu }}_{{\underline{\kappa }}_{j}}^{h}+{\underline{\mu }}_{{\underline{\kappa }}_{j}}^{h}\right)}\right) \end{array}$$

where, $n_{\kappa }$ and *Y* are the number of slices and inputs. From (21), $G_{1}\left(\mu |\Xi_{1}\right)$ and $G_{2}\left(\mu |\Xi_{2}\right)$ we have:

(22) $$G\left(\mu |\Xi_{y_{1}}\right)=\Xi_{}^{T}\mu ,$$

where,

(23) $$\begin{array}{c} \Xi_{}^{T}=\left[{\underline{\Xi }}_{1,1},\ldots ,{\underline{\Xi }}_{1,n_{r}},\ldots ,{\underline{\Xi }}_{Y,1},\ldots ,{\underline{\Xi }}_{Y,n_{r}}\right.,\\ \left.\;\;\;\;\;\;\;\;\;\;\;\;\;\;{\bar{\Xi }}_{1,1},\ldots ,{\bar{\Xi }}_{1,n_{r}},\ldots ,{\bar{\Xi }}_{1,1},\ldots ,{\bar{\Xi }}_{1,n_{r}}\right], \end{array}$$

(24) $$\begin{array}{c} \mu_{}^{T}=\frac{0.5}{\sum_{j=1}^{\Lambda }{\bar{\kappa }}_{j}}\left[\frac{{\underline{\kappa }}_{1}\left({\bar{\mu }}_{{\underline{\kappa }}_{1}}^{1}+{\underline{\mu }}_{{\underline{\kappa }}_{1}}^{1}\right)}{\sum_{h=1}^{Y}\left({\bar{\mu }}_{{\underline{\kappa }}_{j}}^{1}+{\underline{\mu }}_{{\underline{\kappa }}_{j}}^{1}\right)},\ldots ,\frac{{\underline{\kappa }}_{n_{\kappa }}\left({\bar{\mu }}_{{\underline{\kappa }}_{n_{\kappa }}}^{1}+{\underline{\mu }}_{{\underline{\kappa }}_{n_{\kappa }}}^{1}\right)}{\sum_{h=1}^{Y}\left({\bar{\mu }}_{{\underline{\kappa }}_{j}}^{1}+{\underline{\mu }}_{{\underline{\kappa }}_{j}}^{1}\right)}\right.,\\ \;\;\;\;\;\;\frac{{\underline{\kappa }}_{1}\left({\bar{\mu }}_{{\underline{\kappa }}_{1}}^{Y}+{\underline{\mu }}_{{\underline{\kappa }}_{1}}^{Y}\right)}{\sum_{h=1}^{Y}\left({\bar{\mu }}_{{\underline{\kappa }}_{j}}^{1}+{\underline{\mu }}_{{\underline{\kappa }}_{j}}^{1}\right)},\ldots ,\frac{{\underline{\kappa }}_{n_{\kappa }}\left({\bar{\mu }}_{{\underline{\kappa }}_{n_{\kappa }}}^{Y}+{\underline{\mu }}_{{\underline{\kappa }}_{n_{\kappa }}}^{Y}\right)}{\sum_{h=1}^{Y}\left({\bar{\mu }}_{{\underline{\kappa }}_{j}}^{1}+{\underline{\mu }}_{{\underline{\kappa }}_{j}}^{1}\right)},\\ \;\;\;\;\;\;\;{\bar{\kappa }}_{1}\frac{\left({\bar{\mu }}_{{\bar{\kappa }}_{1}}^{1}+{\underline{\mu }}_{{\bar{\kappa }}_{1}}^{1}\right)}{\sum_{h=1}^{Y}\left({\bar{\mu }}_{{\bar{\kappa }}_{j}}^{h}+{\underline{\mu }}_{{\bar{\kappa }}_{j}}^{h}\right)},\ldots ,{\bar{\kappa }}_{n_{\kappa }}\frac{\left({\bar{\mu }}_{{\bar{\kappa }}_{n_{\kappa }}}^{1}+{\underline{\mu }}_{{\bar{\kappa }}_{n_{\kappa }}}^{1}\right)}{\sum_{h=1}^{Y}\left({\bar{\mu }}_{{\bar{\kappa }}_{j}}^{h}+{\underline{\mu }}_{{\bar{\kappa }}_{j}}^{h}\right)},\\ \left.\;\;\;\;\;\;{\bar{\kappa }}_{1}\frac{\left({\bar{\mu }}_{{\bar{\kappa }}_{1}}^{Y}+{\underline{\mu }}_{{\bar{\kappa }}_{1}}^{Y}\right)}{\sum_{h=1}^{Y}\left({\bar{\mu }}_{{\bar{\kappa }}_{j}}^{h}+{\underline{\mu }}_{{\bar{\kappa }}_{j}}^{h}\right)},\ldots ,{\bar{\kappa }}_{n_{\kappa }}\frac{\left({\bar{\mu }}_{{\bar{\kappa }}_{n_{\kappa }}}^{Y}+{\underline{\mu }}_{{\bar{\kappa }}_{n_{\kappa }}}^{Y}\right)}{\sum_{h=1}^{Y}\left({\bar{\mu }}_{{\bar{\kappa }}_{j}}^{h}+{\underline{\mu }}_{{\bar{\kappa }}_{j}}^{h}\right)}\right], \end{array}$$

The rules are optimized as follows:

(25) $$\Xi \left(t+1\right)=\Xi \left(t\right)+\gamma \mu \left(t\right)e\left(t\right)$$

where, *e* represents tracking error and 0≤γ<1.

## 4. Data Driven Control System

From (5), the basic controller is considered as:

(26) $$\eta_{z}={\ddot{r}}_{d_{z}}-G_{z}\left(x|\Xi_{r_{z}}\right)-\iota_{1}{\dot{e}}_{z}-\iota_{2}e_{z}$$

where, $G_{z}\left(x|\Xi_{r_{z}}\right)$ is suggested FLS, $r_{d_{z}}$ is the desired signal, and $\iota_{1}$ and $\iota_{2}$ are constant. Then tracking error dynamics becomes:

(27) $${\ddot{e}}_{z}=-\iota_{1}{\dot{e}}_{z}-\iota_{2}e_{z}-\varepsilon_{z}-u_{z}$$

where, $\varepsilon_{z}$ represents the approximation error. The linearized equations of error system are considered as:

(28) $$\begin{array}{cc} \; & e_{z}\left(k+1\right)=Ae_{z}\left(k\right)+Bu_{z}\left(k\right) \end{array}$$

As mentioned before, we consider a linear discrete time system to model the error system where the system information are fully unknown. Therefore, the aim of this section is to construct a feedback control system no the basis of limited collected data from previous operation of the system, in order to steer the error to the origin. In this regard, a feedback controller $u_{z}\left(k\right)=Fe_{z}\left(k\right)$ is considered and we have to formulate the gain *F* based on data (without identifying the matrices A,B). During the operation of the system, we collect $\tau_{k}$ samples from the input and error as the sequences of $u_{z}\left(0\right),u_{z}\left(1\right),\ldots ,u_{z}\left(\tau_{k}-1\right)$ and $e_{z}\left(0\right),e_{z}\left(1\right),\ldots ,e_{z}\left(\tau_{k}-1\right)$. These are organized as

(29) $$\begin{array}{cc} U_{z\left[0,\tau_{k}\right]} & =[u_{z}\left(0\right)\;u_{z}\left(1\right)\;\ldots \;u_{z}\left(\tau_{k}-1\right)] \end{array}$$

(30) $$\begin{array}{cc} E_{z\left[0,\tau_{k}\right]} & =[e_{z}\left(0\right)\;e_{z}\left(1\right)\;\ldots \;e_{z}\left(\tau_{k}-1\right)] \end{array}$$

(31) $$\begin{array}{cc} E_{z\left[1,\tau_{k}-1\right]} & =[e_{z}\left(1\right)\;e_{z}\left(2\right)\;\ldots \;e_{z}\left(\tau_{k}-1\right)] \end{array}$$

**Lemma** **1.** *Let $C\in R^{\tau_{k}\times n}$ satisfies $I=E_{z\left[0,\tau_{k}\right]}C$. Then, the error system (28) with the state feedback controller $u_{z}\left(k\right)=Fe_{z}\left(k\right)$ which is formulated as $F=U_{z\left[0,\tau_{k}\right]}C$ has the following equivalent representation*

(32) $$\begin{array}{c} e_{z}\left(k+1\right)=E_{z\left[1,\tau_{k}\right]}Ce_{z}\left(k\right) \end{array}$$

**Proof.**  Applying the state feedback controller $u_{z}\left(k\right)=Fe_{z}\left(k\right)$ to the error system (28) and based on $I=E_{z\left[0,\tau_{k}\right]}C$, one can achieve that

(33) $$\begin{array}{cc} e_{z}\left(k+1\right) & =\left(A.I+BF\right)e_{z}\left(k\right)\\ \; & =\left(AE_{z\left[0,\tau_{k}\right]}C+BU_{z\left[0,\tau_{k}\right]}C\right)e_{z}\left(k\right) \end{array}$$

Since the error data in (31) satisfies $E_{z\left[1,\tau_{k}\right]}=AE_{z\left[0,\tau_{k}\right]}+BU_{z\left[0,\tau_{k}\right]}$, Lemma 1 is proved.

□

In the next part, sufficient condition in the framework of LMI will be proposed for the stabilization of the error system (32).

**Theorem** **1.** *If there exist a matrix R such that the following LMI holds*

(34) $$\begin{array}{c} \left[\begin{array}{cc} E_{z\left[0,\tau_{k}\right]}R & E_{z\left[1,\tau_{k}\right]}R\\ {}* & E_{z\left[0,\tau_{k}\right]}R \end{array}\right]>0 \end{array}$$

*Then, the error system *(28)* is asymptotically stable and the direct data-driven controller is computed via $u_{z}\left(k\right)=U_{z\left[0,\tau_{k}\right]}R{\left(E_{z\left[0,\tau_{k}\right]}R\right)}^{-1}e_{z}\left(k\right)$.*

**Proof.**  Since (32) is the data-based closed-loop representation of the error system (28), the asymptotical stability of (32) should be guaranteed. For this purpose, the Lyapunov function is considered as $V\left(k\right)={e_{z}}^{T}\left(k\right)Pe_{z}\left(k\right)$.

The time difference of V(k) is as follows

(35) $$\begin{array}{cc} \Delta V(k) & ={e_{z}}^{T}\left(k+1\right)Pe_{z}\left(k+1\right)-{e_{z}}^{T}\left(k\right)Pe_{z}\left(k\right)\\ \; & ={e_{z}}^{T}\left(k\right)\left(E_{z\left[1,\tau_{k}\right]}RP^{-1}R^{T}E_{z\left[1,\tau_{k}\right]}^{T}\right)e_{z}\left(k\right)-{e_{z}}^{T}\left(k\right)Pe_{z}\left(k\right) \end{array}$$

where the change of variable R=CP is employed. Regarding Lemma 1, one can achieve that $P=E_{z\left[0,\tau_{k}\right]}R$. Now, applying Schur Complement Lemma to the inequality ΔV(k)<0 results in the LMI (34). Furthermore the gain of the direct data-driven controller is computed as $F=U_{z\left[0,\tau_{k}\right]}C=U_{z\left[0,\tau_{k}\right]}RP^{-1}=U_{z\left[0,\tau_{k}\right]}R{\left(E_{z\left[0,\tau_{k}\right]}R\right)}^{-1}$. This completes the proof. □

## 5. Stabilization with Noisy Data

Consider the error system (28), but in this system the only measurable signal is

(36) $$\begin{array}{c} e_{n}\left(k\right)=e_{z}\left(k\right)+n\left(k\right) \end{array}$$

where n(k) is an unknown measurement noise with no particular statistics on the noise. The aim here is to construct a stabilizing control technique on the basis of the open-loop measurable noisy data $e_{n}\left(k\right)$. Defining the following data sequences

(37) $$\begin{array}{cc} N_{\left[0,\tau_{k}\right]} & =[n\left(0\right)\;n\left(1\right)\;\ldots \;n\left(\tau_{k}-1\right)] \end{array}$$

(38) $$\begin{array}{cc} N_{\left[1,\tau_{k}\right]} & =[n\left(1\right)\;n\left(2\right)\;\ldots \;n\left(\tau_{k}\right)] \end{array}$$

in which $n\left(k\right),k=0,\ldots ,\tau_{k}$ are noise samples collected during the experiment. Moreover, one has

(39) $$\begin{array}{cc} E_{n\left[0,\tau_{k}\right]} & =E_{z\left[0,\tau_{k}\right]}+N_{\left[0,\tau_{k}\right]} \end{array}$$

(40) $$\begin{array}{cc} E_{n\left[1,\tau_{k}\right]} & =E_{z\left[1,\tau_{k}\right]}+N_{\left[1,\tau_{k}\right]} \end{array}$$

**Assumption** **1.** *The matrices $\left[\begin{array}{c} U_{\left[0,\tau_{k}\right]}\\ E_{n\left[0,\tau_{k}\right]} \end{array}\right],E_{n\left[1,\tau_{k}\right]}$ are full ranked.*

**Assumption** **2.** *For δ>0 and $S_{n\left[0,\tau_{k}\right]}=AN_{\left[0,\tau_{k}\right]}-N_{\left[1,\tau_{k}\right]}$, the following inequality holds*

(41) $$\begin{array}{c} S_{n\left[0,\tau_{k}\right]}S_{n\left[0,\tau_{k}\right]}^{T}\leq \delta E_{n\left[1,\tau_{k}\right]}E_{n\left[1,\tau_{k}\right]}^{T} \end{array}$$

The Theorem 2, provides sufficient conditions for the stabilization of (28) with noisy collected data.

**Theorem** **2.** *Assume that Assumptions 1 and 2 hold and there exists a matrix R and a scalar μ>0 such that $\delta <\frac{\mu^{2}}{\left(4+2\mu \right)}$ and the following LMIs hold*

(42) $$\begin{array}{cc} \; & \left[\begin{array}{cc} E_{n\left[0,\tau_{k}\right]}R-\mu E_{n\left[1,\tau_{k}\right]}E_{n\left[1,\tau_{k}\right]}^{T} & E_{n\left[1,\tau_{k}\right]}R\\ {}* & E_{n\left[0,\tau_{k}\right]}R \end{array}\right]>0 \end{array}$$

(43) $$\begin{array}{cc} \; & \left[\begin{array}{cc} I & R\\ {}* & E_{n\left[0,\tau_{k}\right]}R \end{array}\right]>0 \end{array}$$

*Then, the error system *(28)* is asymptotically stable and the gain of the data-based controller is designed under noisy collected data via $F=U_{\left[0,\tau_{k}\right]}R{\left(E_{n\left[0,\tau_{k}\right]}R\right)}^{-1}$.*

**Proof.**  From Lemma 1, Equations (39) and (40), the following results can be concluded

(44) $$\begin{array}{cc} e_{n}\left(k+1\right) & =\left(A+BF\right)e_{n}\left(k\right)=\left(AE_{n\left[0,\tau_{k}\right]}C+BU_{\left[0,\tau_{k}\right]}C\right)e_{n}\left(k\right)\\ \; & =\left(A\left(E_{\left[0,\tau_{k}\right]}+N_{\left[0,t_{k}\right]}\right)C+BU_{\left[0,t_{k}\right]}C\right)e_{n}\left(k\right)\\ \; & =\left(E_{n\left[1,\tau_{k}\right]}+S_{n\left[0,\tau_{k}\right]}\right)Ce_{n}\left(k\right) \end{array}$$

Consider Lyapunov candidate as $V\left(k\right)={e_{n}}^{T}\left(k\right)Pe_{n}\left(k\right)$. The time difference of V(k) is:

(45) $$\begin{array}{cc} \; & \Delta V\left(k\right)={e_{n}}^{T}\left(k+1\right)Pe_{n}\left(k+1\right)-{e_{n}}^{T}\left(k\right)Pe_{n}\left(k\right)\\ \; & ={e_{n}}^{T}\left(k\right)\left(\left(E_{n\left[1,\tau_{k}\right]}+S_{n\left[0,\tau_{k}\right]}\right)R{\left(E_{n\left[0,\tau_{k}\right]}R\right)}^{-1}R^{T}\right.\\ \; & \left.{\left(E_{n\left[1,\tau_{k}\right]}+S_{n\left[0,\tau_{k}\right]}\right)}^{T}-E_{n\left[0,\tau_{k}\right]}R\right)e_{n}\left(k\right) \end{array}$$

in which R=CP and $E_{n\left[0,\tau_{k}\right]}R=P$ are utilized. From (45), ΔV(k)<0 results in ${e_{n}}^{T}\left(k\right)\Omega e_{n}\left(k\right)<0$, where

(46) $$\begin{array}{cc} \Omega & =\left(1+\rho \right)E_{n\left[1,\tau_{k}\right]}R{\left(E_{n\left[0,\tau_{k}\right]}R\right)}^{-1}{\left(E_{n\left[1,\tau_{k}\right]}R\right)}^{T}\\ \; & +\left(1+\rho^{-1}\right)S_{n\left[1,\tau_{k}\right]}R{\left(E_{n\left[0,\tau_{k}\right]}R\right)}^{-1}{\left(S_{n\left[0,\tau_{k}\right]}R\right)}^{T}-E_{n\left[0,\tau_{k}\right]}R \end{array}$$

in which ρ>0. Regarding LMI (43), one has

(47) $$\begin{array}{cc} \Omega & <-\mu E_{n\left[1,\tau_{k}\right]}E_{n\left[1,\tau_{k}\right]}^{T}+\rho E_{n\left[1,\tau_{k}\right]}E_{n\left[1,\tau_{k}\right]}^{T}\\ \; & +\left(1+\rho^{-1}\right)S_{n\left[0,\tau_{k}\right]}S_{n\left[0,\tau_{k}\right]}^{T} \end{array}$$

Now, choosing $\rho =\frac{\mu }{2}$ and considering $\delta <\frac{\mu^{2}}{4+2\mu }$ imply that the upper bound of Ω in (47) is negative. Therefore, the proof is completed. □

**Remark** **1.** *The advantages of the control scheme are that: (1) The uncertainties in dynamics of the MEMS-G are online identified and there is no dependence on the predefined mathematical equations. (2) The data of tracking error is collected and then a data-driven compensator is designed. (3) The asymptotic stability is proved in two normal and noisy conditions.*

## 6. Simulations

**Figure 4:**
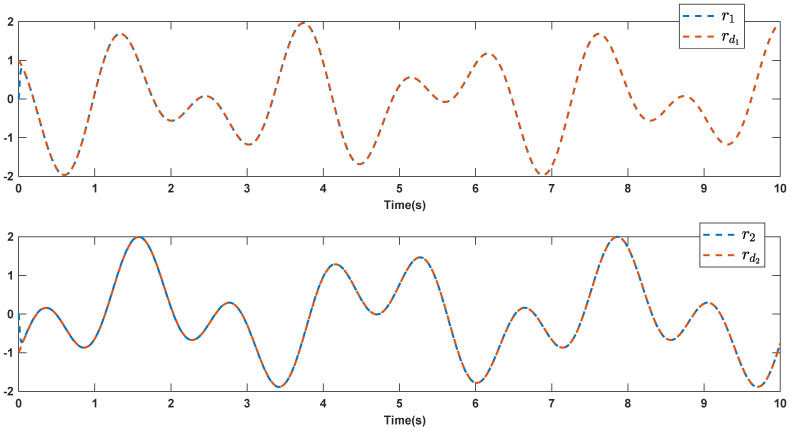
Output signals.

**Figure 5:**
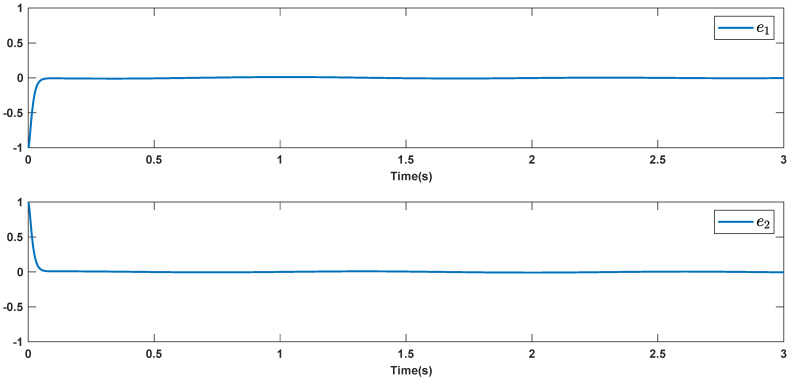
Tracking errors.

**Figure 6:**
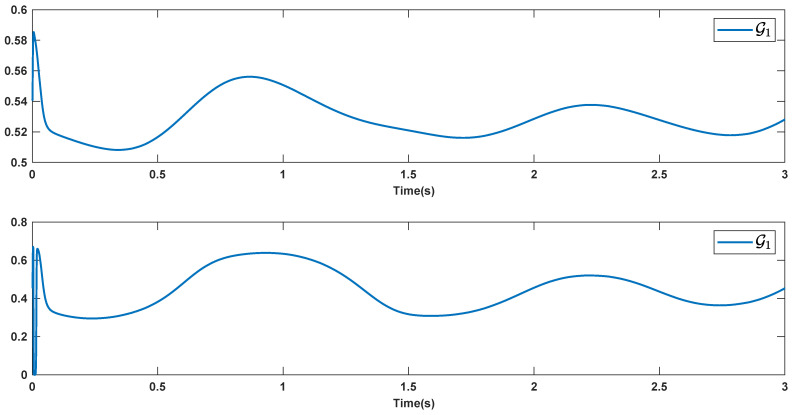
Estimated signals.

**Figure 7:**
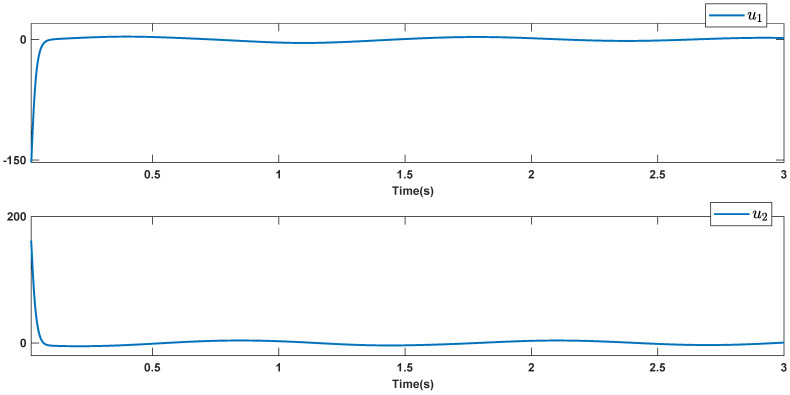
Control signals.

**Figure 8:**
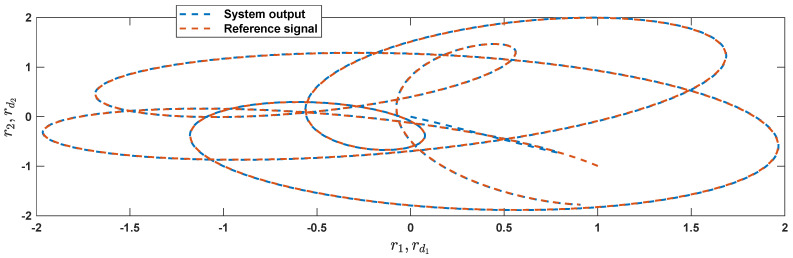
Phase portrait.

The reference signals $r_{d_{1}}$ and $r_{d_{2}}$ are considered to be cos(5t)−sin(3t) and sin(5t)−cos(2t−0.1). The initial conditions are as: $r_{1}\left(0\right)=-0.71$ and $r_{2}\left(0\right)=-0.91$. The simulation parameters are given in Table 1. The trajectories of $r_{i},\;i=1,2$ are depicted in Figure 4 that show good tracking. We see that $r_{i},\;i=1,2$ are approached to the references $r_{d_{i}},\;i=1,2$ at a finite time with no overshoots. The trajectories of $e_{1}$ and $e_{2}$ in Figure 5 show that the settling time is desired and error signals are reached to the zero level at a short time. The estimated signals, control signals and phase portrait are given in Figure 6, Figure 7 and Figure 8, respectively. A strong synchronization in observed in Figure 8.

For further evaluation, a comparison is presented with backstepping SMC (BSMC) [46], FLS based SMC (FSMC) [47] and fractional-order neural-based controller (FNC) [48]. Table 2, presents the RMSE comparison results. We see that the designed data-driven controller gives a better result. It worth to mention that the dynamics in our controller is fully unknown.

**Remark** **2.** *One of the main properties of the suggested controller is that the uncertainties are tackled by a powerful estimation scheme based on T3-FLSs. On the other hand, a data-driven compensator helps that the tracking error to be reduced. Then we see from simulation results that the tracking errors are well reached to zero level at a good finite time.*

**Figure 9:**
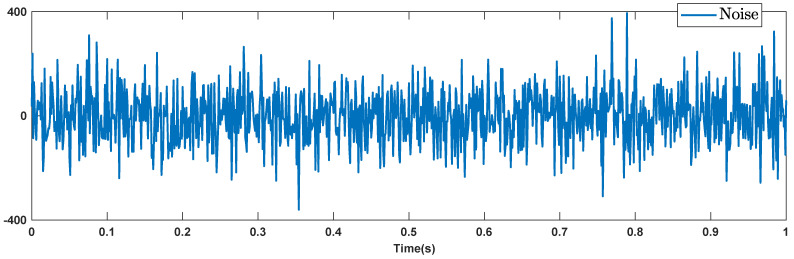
High noise as dynamic perturbation.

**Figure 10:**
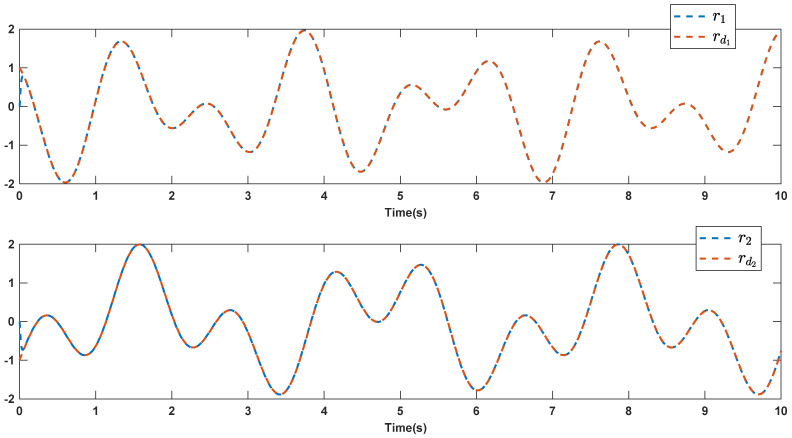
Output signals in the presence of high noisy condition.

**Figure 11:**
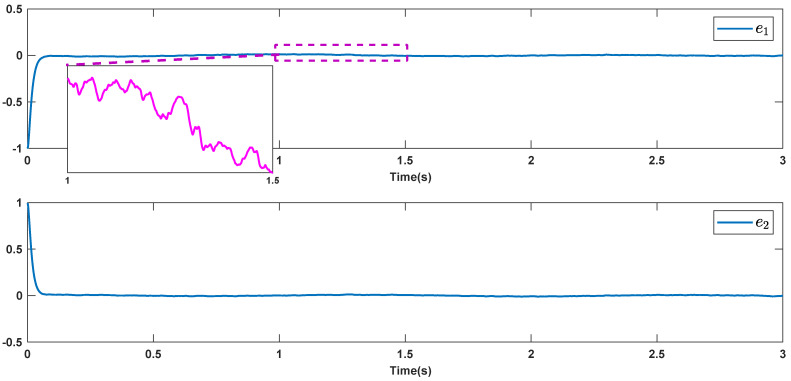
Tracking errors in the presence of high noisy condition.

**Figure 12:**
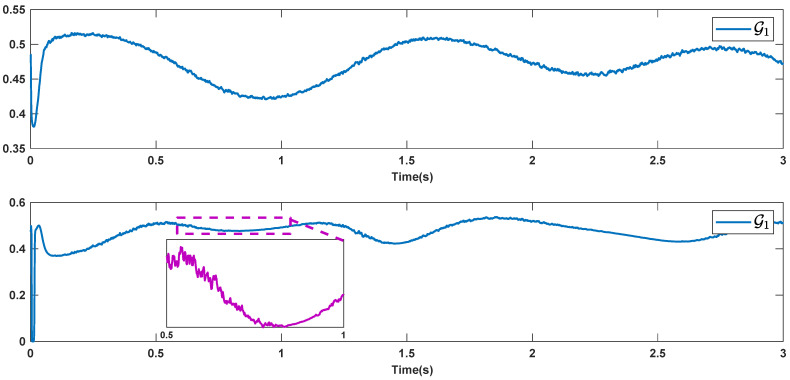
Estimated signals in the presence of high noisy condition.

**Figure 13:**
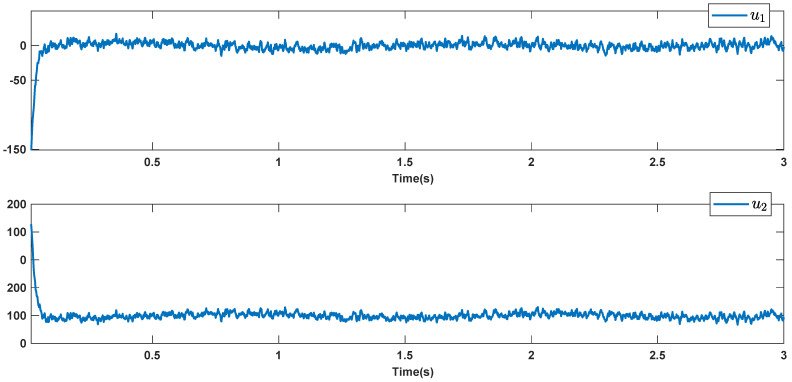
Control signals in the presence of high noisy condition.

To better examine the robustness against the hard noisy condition, the disturbances are considered to be noise with noise-power 10 which is shown in Figure 9. The trajectories of output signals, tracking errors, estimated signals, and control signals are given in Figure 10, Figure 11, Figure 12 and Figure 13. We see that the suggested approach results in a good robust performance. The output signals well track the references in the presence of high noisy condition and unknown dynamics.

## 7. Conclusions

This study proposes a novel data-driven control scheme for MEMS gyroscopes (MEMS-Gs) based on type-3 FLSs. The suggested FLS by a non-singleton fuzzification is designed to compensate the perturbations, uncertainties and measurement errors. The data-driven control technique by guaranteed stability is proposed to develop the stability, accuracy and robustness. The suggested controller is applied on a case-study gyroscope. Besides the fully unknown dynamics, a measurement error and dynamic perturbation are also considered as Gaussian noise. The simulations and comparisons with some conventional approaches, show that the suggested data-driven control scheme results in better accuracy.

## Figures and Tables

**Table 1:** Control parameters.

Parameters	Values	Equations
$\bar{\kappa }$	0.5 0.8 1	(12) and (13)
$\underline{\kappa }$	0 0.3 0.7	(14) and (15)
$c_{\Lambda_{1}^{1}}$	−1	(12)–(15)
$c_{\Lambda_{1}^{2}}$	1	(12)–(15)
$c_{\Lambda_{2}^{1}}$	−1	(12)–(15)
$c_{\Lambda_{2}^{2}}$	1	(12)–(15)
$\iota_{1}$	100	(27)
$\iota_{2}$	20	(27)

**Table 2:** Comparison results.

Controller	$e_{1}$	$e_{2}$
BSMC	7.4207	11.1113
FSMC	5.3741	10.3440
FNC	6.1931	7.0221
Proposed Controller	3.4442	3.4373

## Data Availability

The study do not report any data.
